# Prominence of the Metric System

**Published:** 1887-11

**Authors:** 


### Prominence of the Metric System.

Wm. G. Eggleston, in the *Journal of the American Medical Association*, says: "The truth concerning the neglect of the metric system in this country is that, in the first place, it is not properly taught in schools, nor are decimals properly taught; in the second place it is a little trouble to learn something new and strange, and our fathers got on pretty well with their system. I will venture the assertion that there are not half a dozen good chemists in this country or any other who use the apothecary's weight in their work. All books on examination of urine, etc., are written entirely in the metric system. If there were no other reason for adopting the metric system, the fact that it is used by the large majority of medical and scientific men—and has been found by them to be uniform, generally applicable, convenient, simple, safe, and scientific in character—would be alone sufficient."

